# Renal Hyperfiltration and Systemic Blood Pressure in Patients with Uncomplicated Type 1 Diabetes Mellitus

**DOI:** 10.1371/journal.pone.0068908

**Published:** 2013-07-04

**Authors:** Gary K. Yang, David M. Maahs, Bruce A. Perkins, David Z. I. Cherney

**Affiliations:** 1 Faculty of Medicine, University of Toronto, Toronto, Ontario, Canada; 2 Barbara Davis Center for Childhood Diabetes, University of Colorado Denver, Aurora, Colorado, United States of America; 3 Department of Medicine, Division of Endocrinology, Toronto General Hospital, University of Toronto, Toronto, Ontario, Canada; 4 Department of Medicine, Division of Nephrology, Toronto General Hospital, University of Toronto, Toronto, Ontario, Canada; Children’s Hospital Boston/Harvard Medical School, United States of America

## Abstract

**Background:**

Patients with type 1 diabetes mellitus (DM) and renal hyperfiltration also exhibit systemic microvascular abnormalities, including endothelial dysfunction. The effect of renal hyperfiltration on systemic blood pressure (BP) is less clear. We therefore measured BP, renal hemodynamic function and circulating renin angiotensin aldosterone system (RAAS) mediators in type 1 DM patients with hyperfiltration (n = 36, DM-H, GFR≥135 ml/min/1.73 m^2^) or normofiltration (n = 40, DM-N), and 56 healthy controls (HC). Since renal hyperfiltration represents a state of intrarenal RAAS activation, we hypothesized that hyperfiltration would be associated with higher BP and elevated levels of circulating RAAS mediators.

**Methods:**

BP, glomerular filtration rate (GFR - inulin), effective renal plasma flow (paraaminohippurate) and circulating RAAS components were measured in DM-H, DM-N and HC during clamped euglycemia (4–6 mmol/L). Studies were repeated in DM-H and DM-N during clamped hyperglycemia (9–11 mmol/L).

**Results:**

Baseline GFR was elevated in DM-H vs. DM-N and HC (167±6 vs. 115±2 and 115±2 ml/min/1.73 m^2^, p<0.0001). Baseline systolic BP (SBP, 117±2 vs. 111±2 vs. 109±1, p = 0.004) and heart rate (76±1 vs. 67±1 vs. 61±1, p<0.0001) were higher in DM-H vs. DM-N and HC. Despite higher SBP in DM-H, plasma aldosterone was lower in DM-H vs. DM-N and HC (42±5 vs. 86±14 vs. 276±41 ng/dl, p = 0.01). GFR (p<0.0001) and SBP (p<0.0001) increased during hyperglycemia in DM-N but not in DM-H.

**Conclusions:**

DM-H was associated with higher heart rate and SBP values and an exaggerated suppression of systemic aldosterone. Future work should focus on the mechanisms that explain this paradox in diabetes of renal hyperfiltration coupled with systemic RAAS suppression.

## Introduction

Diabetes mellitus (DM) is the leading cause of end-stage renal disease, which ultimately requires dialysis or transplantation. Although the physiological factors that cause early diabetic renal injury remain incompletely understood, renal hyperfiltration has been associated with microalbuminuria and a decline in glomerular filtration rate (GFR) in some but not all studies [1], [2], [3]. Inconsistencies around the role of hyperfiltration in the pathogenesis of diabetic nephropathy may relate to differences in GFR measurement methods, variable definitions of hyperfiltration and patient-related factors such as diet and ambient hyperglycemia [4], [5], [6]. Nevertheless, hyperfiltration may contribute to progression of kidney disease and has been attributed to alterations in renal hemodynamic function and tubuloglomerular feedback [4]. As such, a better understanding of the factors that contribute to the development of diabetic nephropathy, including hyperfiltration, is clearly warranted.

For hemodynamic factors that contribute to renal hyperfiltration, the renin angiotensin aldosterone system (RAAS) has been the most widely studied because of the availability of medications that block the RAAS [7], [8]. RAAS activation is associated with high intraglomerular pressure in animals and humans with type 1 DM, and the use of RAAS inhibitors reduces intraglomerular pressure in animals and hyperfiltration in humans [8], [9]. The etiology of the intrarenal RAAS characteristic of DM is incompletely understood but has been commonly attributed to the effect of ambient hyperglycemia on angiotensinogen generation [10]. Hyperglycemia does not, however, explain why the intrarenal RAAS is preferentially activated in some patients and not others, including those with renal hyperfiltration [4].

In addition to abnormalities in the kidney, renal hyperfiltration is associated with changes in systemic vascular function, including endothelial dysfunction and low levels of circulating nitric oxide bioavailability [11], [12]. These subclinical abnormalities in endothelial function may be of particular importance in hyperfiltering individuals, who may also exhibit modest elevations in blood pressure within the normal range, including impaired nocturnal blood pressure dipping patterns [13], [14]. Elevations in systemic blood pressure within the “normal range” may in turn be important because of the increased risk of microalbuminuria and retinopathy associated with these changes [15], [16], [17]. Despite what is known about the RAAS in the kidney in hyperfiltering patients, less is known about the interaction between blood pressure, levels of circulating RAAS mediators and renal hyperfiltration, as well as the effect of ambient glycemia on these factors.

Accordingly, we examined a cohort of young patients with type 1 DM with either renal hyperfiltration (DM-H, GFR≥135 ml/min/1.73 m^2^) or normofiltration during clamped euglycemia (DM-N, GFR<135 ml/min/1.73 m^2^) [12], [18], and compared them to a similar cohort of healthy control (HC) participants. Participants were studied under these controlled baseline glycemic conditions to avoid the confounding effects of different ambient glucose levels on GFR [18], [19]. Since DM-H exhibit intrarenal RAAS activation in previous work [8], we hypothesized that DM-H would exhibit higher blood pressure values compared with DM-N and HC, and that higher blood pressure values would be associated with elevated levels of circulating RAAS mediators. We also studied diabetic participants under clamped hyperglycemic conditions because of the activating effect of hyperglycemia on the RAAS. We hypothesized that hyperglycemia would result in blunted hemodynamic effects in DM-H due to baseline activation of the RAAS.

## Methods

A total of 56 healthy control (HC) and 76 participants with uncomplicated type 1 DM (36 normofiltering – “DM-N” and 40 hyperfiltering – “DM-H”) were included in this study. Hyperfiltration was defined using the usual definition of a GFR ≥135 ml/min/1.73 m^2^ [2]. Inclusion criteria were: duration of type 1 DM >1 year, ages 18–30 years, blood pressure <140/90 mmHg, normoalbuminuria on a 24 hour urine collection, no history of renal disease or macrovascular disease or regular medications other than insulin, including oral contraceptives. Patients taking RAAS blocking agents were excluded. Visits for female subjects were scheduled to coincide with the follicular phase of the menstrual cycle, determined by cycle day and measurement of 17ß-estradiol levels. DM participants were studied under both clamped euglycemic (Day 1, 4–6 mmol/L) and hyperglycemic (Day 2, 9–11 mmol/L) conditions and HC were studied under normoglycemic conditions. The Research Ethics Board at the University Health Network approved the protocol and all subjects gave written informed consent.

In order to maintain suppression of endogenous RAS activity and to standardize study conditions, subjects adhered to a high sodium (>140 mmol/day) and moderate protein (<1.5 g/kg/day) diet during the 7-day period before each experiment, as described previously [20]. On two consecutive days, brachial artery blood pressure (Critikon, Tampa, Florida, USA) and renal hemodynamic parameters were obtained after a 6-hour modified clamp, during clamped euglycemia (and hyperglycemia [20]. On the euglycemic day, renal hemodynamic functions (GFR and effective renal plasma flow - ERPF) were estimated using inulin and paraaminohippurate (PAH) clearance techniques, respectively. In brief, a 16-gauge peripheral venous cannula was inserted into the left antecubital vein for infusion of glucose and insulin and a second cannula was inserted for blood sampling more distally. Blood glucose was measured every 5–10 minutes and the insulin infusion was adjusted to maintain euglycemia. After the desired level of ambient glycemia was maintained for 6 hours, a third intravenous line was inserted into the right arm and was connected to a syringe infusion pump for administration of inulin and PAH. After collecting blood for inulin and PAH blanks, a priming infusion containing 25% inulin (60 mg/kg) and 20% PAH (8 mg/kg) was administered. Thereafter, inulin and PAH were infused continuously at a rate calculated to maintain their respective plasma concentrations constant at 20 and 1.5 mg/dl. After a 90 minute equilibration period, blood was collected for inulin, PAH and hematocrit (HCT) measurements. Blood was further collected at 30 minutes and 60 minutes for inulin and PAH measurements. GFR and ERPF were estimated by steady state infusion of inulin and PAH, respectively [21].

After the desired level of clamped euglycemia or hyperglycemia was achieved, baseline blood samples were also collected for the measurement of angiotensinogen, plasma renin concentration (PRC), plasma renin activity (PRA), angiotensin II and aldosterone. All measurements and samples were taken in the same warm (25°C) temperature-controlled rooms after 10 min of rest in the supine position.

### Sample Collection and Analytical Methods

Blood samples collected for inulin and PAH determinations were immediately centrifuged at 3000 rpm for 10 minutes at 4°C. Plasma was separated, placed on ice, and then stored at −70°C before the assay. Inulin and PAH were measured in serum by colorimetric assays using anthrone and N- (1-naphthyl) ethylenediamine respectively [22], [23], [24]. The mean of two baseline clearance periods represents GFR and ERPF, expressed as per 1.73 m^2^. Renal blood flow (RBF) was derived using ERPF/(1-hematocrit) and renal vascular resistance (RVR) was derived by dividing the MAP by the RBF, as described previously by our group and by others [20], [25]. All renal hemodynamic measurements were adjusted for body surface area [22], [24].

Ang II was measured by radioimmunoassay. Blood was collected into pre-chilled tubes containing EDTA and angiotensinase inhibitor (0.1 ml Bestatin Solution, Buhlmann Laboratories, Switzerland). After centrifugation, plasma samples were stored at −70°C until analysis. On the day of analysis plasma samples were extracted on phenylsilysilica columns. A competitive radioimmunoassay kit supplied by Buhlmann Laboratories AG (Switzerland) was used to measure the extracted Ang II. Aldosterone was measured by radioimmunoassay, using the Coat-A-Count system. Active plasma renin was measured by 2-site immunoradiometric assay where two monoclonal antibodies to human active renin are used. One antibody was coupled to biotin while the second was radiolabeled for detection. The sample containing active renin was incubated simultaneously with both antibodies to form a complex. The radioactivity of this complex was directly proportional to the amount of immunoreactive renin present in the sample. PRA was measured with a radioimmunoassay kit (GammaCoat® Plasma Renin Activity ^125^I RIA Kit, CA-1533, Diasorin, Stillwater, Minnesota 55082–0285, USA). PRA determination involves an initial incubation of plasma to generate angiotensin I, followed by quantitation of angiotensin I by radioimmunoassay. In the GammaCoat® Plasma Renin Activity ^125^I RIA Kit, the antibody is immobilized onto the lower inner wall of the GammaCoat tube. After incubation of standards, unknown samples and ^125^I angiotensin I in the GammaCoat tube, the reaction mixture is removed by aspiration and the bound tracer counted in a gamma counter. A standard curve is constructed and the concentration of angiotensin I of the unknown sample obtained by interpolation. Angiotensinogen was measured indirectly by converting endogenous angiotensinogen to angiotensin I, then quantitating the amount of angiotensin I by radioimmunoassay. Plasma noradrenaline and adrenaline were measured using standard techniques [26].

Urinary albumin excretion rate was determined from three timed overnight urine collections. Urinary albumin concentration was determined by immunoturbidimetry to determine normoalbuminuria. Hemoglobin A1C was measured by high-performance liquid chromatography, and plasma insulin levels were measured using standard techniques [11].

### Statistical Analysis

Data are presented as mean±SD. We based the sample size calculation on known effects of clamped hyperglycemia on renal hemodynamic function, since these effects are thought to be due to RAAS activation [27]. Under the assumption that the standard deviation of the ΔGFR in response to clamped hyperglycemia will approximate that in our previous work, we anticipated a standard deviation of approximately 16 ml/min/1.73 m^2^ [19]. To detect a 10 ml/min/1.73 m^2^ *within group* change in the GFR response to clamped hyperglycemia, for a two-sided test with alpha 0.05 and 80% power, the sample size should equal a minimum of 24 participants in each group. To assess for baseline *between group* differences, ANOVA was used and significance was defined as p<0.05. To compare responses to hyperglycemia *within* and *between groups*, a repeated measures ANOVA was used and p<0.05 was considered to be significant. All statistical analyses were performed using SPSS v19.0.

## Results

### Demographic Characteristics

Baseline clinical characteristic were similar in the three groups (Table 1). Participants were young and otherwise healthy and urinary Na+ and urea parameters reflected adherence to the prescribed Na^+^ and protein diet. They were also normotensive and normoalbuminuric, and exhibited similar values for weight and body mass index.

**Table 1:** Baseline characteristics, biochemistry and hemodynamic function in healthy control and type 1 diabetes patients during clamped euglycemia (mean±SD).

	HC (n = 56)	DM-N (n = 36)	DM-H (n = 40)
*Baseline parameters*			
Males	26 (46%)	17 (46%)	19 (48%)
Age (years)	23.3±0.3	22.8±0.9	22.3±0.6
Diabetes Duration (years)	N/A	16±1	15±1
Weight (kg) – euglycemia	70.0±2.1	74.7±1.9	71.1±1.8
Weight (kg) – hyperglycemia	N/A	74.9±2.0	71.2±1.9
Height (meters)	1.70±0.01	1.72±0.01	1.70±0.01
Body mass index (kg/m^2^)	24±1	24±1	23±1
HbA1C - % (mmol/mol)	N/A	8.6±0.3	8.4±0.2
Estrogen (pmol/L - in women)	170±56	135±53	168±29
Albumin excretion rate (mg/day)	9.0±1.0	8.3±1.4	9.8±1.6
Sodium excretion (mmol/24 hr)	175±8	178±14	164±7
Protein intake (gram/kg/day)	1.08±0.02	0.98±0.04	0.99±0.04
*Systemic hemodynamic function*			
Heart rate (beats per minute)	61±1	67±1†	76±1* ‡
Systolic blood pressure (mmHg)	109±1	111±2	117±2* ‡
Diastolic blood pressure (mmHg)	63±1	64±1	67±1*
*Renal hemodynamic function*			
Effective renal plasma flow (ml/minute/1.73 m^2^)	629±12	662±14	891±45§
Glomerular filtration rate (ml/minute/1.73 m^2^)	115±2	115±2	167±6§
Filtration fraction	0.18±0.005	0.17±0.01	0.21±0.02
Renal blood flow (ml/minute/1.73 m^2^)	1057±25	1013±23	1419±73§
Renal vascular resistance (mmHg/L/minute)	0.078±0.002	0.081±0.002	0.064±0.004§
GFR Range (ml/minute/1.73 m^2^)	91–138	91–134	135–330
*Circulating neurohormonal factors*			
Aldosterone (ng/dl)	276.1±41.0	85.9±13.5†	41.8±5.2* ‡
ANG II (pg/ml)	12.5±1.2	3.2±0.6†	3.5±0.8*
Renin (pg/ml)	20.0±2.6	5.7±0.7†	5.3±0.5*
Plasma renin activity (ng/mL/h)	1.44±0.16	0.55±0.09†	0.42±0.06*
Angiotensinogen (ng/ml)	2072±290	1211±155†	1280±198*
Norepinephrine (pmol/L)	0.60±0.09	0.70±0.14	0.77±0.08

* p≤0.0001 for HR, SBP, DBP, renin, aldosterone, Ang II, PRA in DM-H vs. HC; p = 0.027 for angiotensinogen in DM-H vs. HC.

† p≤0.01 for HR, renin, aldosterone, Ang II, PRA, angiotensinogen in DM-N vs. HC.

‡ p≤0.01 for HR, SBP and aldosterone in DM-H vs. DM-N.

§ p<0.0001 for between group differences in DM-H vs. DM-N and HC.

### Heart Rate, Blood Pressure, Renal Hemodynamic Function and Circulating RAAS Mediators during Clamped Euglycemia

During clamped euglycemia, heart rate and systolic blood pressure (SBP) were higher in DM-H vs. DM-N and HC, whereas only heart rate was higher in DM-N vs. HC (Table 1). Diastolic blood pressure (DBP) was also higher in DM-H compared with HC. For renal hemodynamic function, GFR, ERPF and RBF were higher and RVR resistance lower in DM-H compared with the other 2 groups. Despite elevated blood pressure values in DM-H, serum aldosterone levels were lowest in DM-H vs. DM-N and HC (42±5 vs. 86±14 vs. 276±41 ng/dl, p = 0.01). PRA, PRC, angiotensin II and angiotensinogen were also markedly suppressed in DM-H and DM-N compared with HC, but differences between DM-H and DM-N did not reach significance (Table 1). There were no significant *between-group* differences in circulating noradrenaline levels (Table 1).

### Heart Rate, Blood Pressure, Renal Hemodynamic Function and Circulating RAAS Mediators during Clamped Hyperglycemia

During clamped hyperglycemia, heart rate remained unchanged in the three groups, while SBP increased significantly in DM-N compared with values during clamped euglycemia, (Table 2, *between-group* effect, p = 0.002). Weight remained unchanged compared to values during clamped euglycemia in both groups (Table 1).

**Table 2:** Biochemistry and hemodynamic function in type 1 diabetes patients during clamped hyperglycemia (mean±SD).

	DM-N (n = 36)	DM-H (n = 40)
*Systemic hemodynamic function*		
Heart rate (beats perminute)	68±2	74±2
Systolic blood pressure (mmHg)	116±2* ¶	117±2
Diastolic blood pressure (mmHg)	65±1	64±1
		
*Renal hemodynamic function*		
Effective renal plasma flow(ml/minute/1.73 m^2^)	725±24*	925±41
Glomerular filtration rate(ml/minute/1.73 m^2^)	143±5* §	175±6
Filtration fraction	0.19±0.01	0.20±0.01
Renal blood flow (ml/minute/1.73 m^2^)	1132±36*	1477±65
Renal vascular resistance(mmHg/L/minute)	0.074±0.001*	0.058±0.002
		
*Circulating neurohormonal factors*		
Aldosterone (ng/dl)	77±19	47±10
ANG II (pg/ml)	3.1±0.5	2.7±0.5
Renin (pg/ml)	3.9±0.6†	3.2±0.4†
Plasma renin activity (ng/mL/h)	0.35±0.06‡	0.26±0.03‡
Angiotensinogen (ng/ml)	1170±150	1154±182
Norepinephrine (pmol/L)	0.53±0.05	0.82±0.25

* p≤0.001 compared to value during clamped euglycemia.

† p≤0.004 vs. level during clamped euglycemia.

‡ p≤0.026 vs. level during clamped euglycemia.

§ p = 0.02 for effect of hyperglycemia on GFR in DM-N vs. DM-H.

¶ p = 0.002 for effect of hyperglycemia on blood pressure in DM-N vs. DM-H.

For renal hemodynamic function, GFR, ERPF and RBF remained higher and RVR resistance lower in DM-H compared with DM-N (Table 2). In response to clamped hyperglycemia, however, the change in GFR was exaggerated in DM-N vs. DM-H (Δ+25±6 vs. Δ+3±7 ml/min/1.73 m^2^, *between-group* effect, p = 0.02).

During clamped hyperglycemia, levels of circulating RAS mediators were numerically lower in DM-H vs. DM-N, but these differences were not significant (Table 2). In response to clamped hyperglycemia, PRA and PRC decreased significantly in both groups, and these changes were not significantly different in DM-H vs. DM-N. Changes in angiotensin II, angiotensinogen and noradrenaline in response to hyperglycemia were not significant in either group.

## Discussion

Renal hyperfiltration in patients with type 1 and type 2 DM is associated with the development of diabetic nephropathy in some studies and has been attributed to activation of neurohormonal factors such as the RAAS and to the influence of tubuloglomerular feedback [4], [28]. In addition to effects in the kidney, renal hyperfiltration is associated with changes in systemic vascular function including endothelial dysfunction and abnormal patterns in nocturnal blood pressure dipping [11], [12], [13]. Whether this *systemic* macrovascular dysfunction in patients with *renal* hyperfiltration is dependent on systemic activation of the RAAS has previously been unknown. Furthermore, the effect of clamped hyperglycemia on blood pressure and circulating RAAS mediators in patients with renal hyperfiltration due to type 1 DM has not been clearly detailed. We hypothesized that DM-H would have higher BP and levels of circulating RAAS mediators, and that DM-H would exhibit blunted hemodynamic responses to clamped hyperglycemia. Our main findings were: 1) Heart rate and blood pressure values were higher in DM-H compared to DM-N and HC; 2) Despite higher blood pressures, circulating RAAS mediators were suppressed in DM-H vs. DM-N; 3) Systemic blood pressure and renal hemodynamic responses to clamped hyperglycemia were exaggerated in DM-N vs. DM-H.

Type 1 DM is associated with an increased risk of hypertension, which contributes to the progression of diabetic nephropathy [29], [30]. Less is known about early, subclinical changes in blood pressure within the normal range in patients with uncomplicated type 1 DM. Because of our study design which used a careful pre-study preparation phase and control of ambient glycemia, we were able to demonstrate relatively small differences in BP between the three groups. In our previous work we demonstrated that brachial artery flow-mediated vasodilatation is impaired in young DM-H patients, suggesting the presence of more generalized abnormalities in vascular function [11], [12], [13]. Consistent with this work was the observation by Pecis et al that nocturnal blood pressure dipping is abnormal and SBP higher on ambulatory blood pressure monitoring in DM-H compared to DM-N and HC [13]. The mechanisms responsible for these BP differences, including the role of the RAAS and ambient hyperglycemia, were not known. In the present study during clamped euglycemia, DM-H also exhibited higher SBP compared with DM-N and HC, and DBP was higher in DM-H vs. HC. These differences were no longer observed during clamped hyperglycemia due to a significant increase in SBP in the DM-N group only. Importantly, weight did not change in either group in response to hyperglycemia, suggesting that changes in SBP were not due to changes in effective circulating volume. Hyperfiltration in our cohort was therefore associated with clinically important ∼5 mmHg differences in SBP compared with DM-N during clamped euglycemia. These blood pressure differences would be clinically silent, since hyperfiltration cannot be reliably detected using creatinine-based estimating equations, making it difficult to identify these small blood pressure increases in DM-H [5], [15]. While clinically silent, similar elevations in systemic blood pressure within the “normal range” are clinically important, due to the associated increased risk of microalbuminuria and retinopathy associated with these changes [15], [16], [17]. In our study, differences in blood pressure in DM-H and DM-N were not on the basis of obvious clinical confounders such as sex, dietary sodium, HbA1c, age, weight or diabetes duration since these factors were similar in the two DM groups.

In addition to blood pressure differences, DM-H exhibited higher heart rate values compared with the other groups. To our knowledge, higher heart rate values in diabetic patients have only been previously described in ambulatory blood pressure monitoring studies, which have also linked higher heart rate values within the normal range to greater risks of developing microalbuminuria and retinopathy [15], [16], [31], [32]. These previous studies did not, however, analyze study participants on the basis of glomerular filtration status. For example, Christiansen et al. demonstrated that young, normoalbuminuric type 1 DM patients have increased heart rate and contractility measures compared with HC, and hypothesized that these changes reflect an increase in systemic sympathetic tone [33]. The present study was not designed to examine autonomic function, aside from circulating noradrenaline levels which were measured and tended to be similar in the three groups. Plasma levels of sympathetic nervous system mediators are not, however, reliable markers of sympathetic activity and this finding should be confirmed using other techniques such as heart rate variability or radiolabeled norepinephrine techniques [34]. Since adrenergic antagonism with beta-blockers have shown beneficial effects on renal hypertrophy in experimental models of DM, as well as reductions in proteinuria in diabetic humans, future work should clarify the role of these agents on early renal hemodynamic abnormalities and blood pressure in patients with uncomplicated type 1 DM [35], [36].

Aside from possible influences of the sympathetic nervous system, the mechanisms responsible for the rise in blood pressure in DM-N patients include extracellular fluid compartment expansion and loss of vasodilatory bioactivity, although volume-mediated changes seem unlikely since weight remained the same on both study days [37], [38]. Activation of the RAAS has also been associated with hypertension, although RAAS-related pathways have been difficult to elucidate in humans with DM because of a phenomenon known as the “paradox of the low renin state in DM” [39]. This physiological principle refers to the observation that systemic levels of RAAS hormones are low in DM, in the context of simultaneous and opposite increased levels in tissue, including the kidney [39]. The RAAS paradox has been described in both animal models [40], [41] and in humans [39] with diabetes and in non-DM patients with chronic kidney disease [42], but the responsible mechanisms for the dissociation between systemic and renal RAAS activity are not known [43]. Specifically, the interaction between renal hyperfiltration and the RAAS paradox in humans, as well as the role of ambient hyperglycemia, have not been described. In the present study, circulating aldosterone levels were lower in DM-H vs. DM-N and HC, suggesting a dose-response relationship. Previous animal and human data have clearly demonstrated that the intrarenal RAAS is activated in the hyperfiltration state, reflected by exaggerated renal hemodynamic responses to RAAS inhibition [8], [9]. Although we did not modulate the RAAS with pharmacological inhibitors or infusions of exogenous angiotensin II in this series of experiments, in the context of this previous work our results suggest that enhanced intrarenal RAAS activation characteristic of DM-H is associated with an exaggerated suppression of circulating RAAS mediators compared with DM-N. It is also of interest that systemic and renal responses to clamped hyperglycemia were attenuated in DM-H compared with DM-N. Miller et al previously demonstrated that the hyperfiltration responses to clamped hyperglycemia are related to intrarenal RAAS activation [10], [27]. Our observation that DM-N exhibited enhanced blood pressure and hyperfiltration responses to hyperglycemia suggests that baseline RAAS activity was relatively low in DM-N vs. DM-H, leading to greater hemodynamic responses with induction of clamped hyperglycemia in DM-N.

Our results may also have implications for future clinical studies which are designed to measure changes in renal function over time. First, because of the influence of ambient hyperglycemia on direct and estimated GFR [5], clinical trials in DM patients should consider controlling glycemic levels at the time that GFR is measured. Such an approach may substantially reduce GFR variability and signal-to-noise ratio, thereby permitting investigators to detect small amplitude but clinically significant changes in GFR over time. Second, our results suggest that future studies related to renal hyperfiltration will have to control for ambient hyperglycemia to avoid “differential misclassification” (i.e. patients who are classified as DM-H, but who have an elevated GFR only because their blood glucose was elevated at the time of GFR measurement).

This study has limitations. First, we did not measure sympathetic activity with gold-standard techniques such as heart rate variability or radiolabeled adrenergic assays. Second, we avoided performing hyperglycemic clamps in HC for two reasons: 1) Hyperglycemic clamps in HC require the use of octreotide, which independently influences plasma renin activity, thereby confounding the study results; 2) Acute hyperglycemia in non-DM HC does not represent a physiological state.

In conclusion, DM-H is associated with an increase in heart rate, higher blood pressure within the normal range, greater suppression of circulating RAAS mediators and blunted responses to clamped hyperglycemia. Due to interactions between the sympathetic nervous system and the intrarenal RAAS in patients with early type 1 DM, future work to investigate mechanisms linking the sympathetic nervous system activity, hyperfiltration and systemic vascular abnormalities may identify therapeutic targets.
